# Loss-of-Function *NUBPL* Mutation May Link Parkinson's Disease to Recessive Complex I Deficiency

**DOI:** 10.3389/fneur.2020.555961

**Published:** 2020-10-29

**Authors:** Peggy S. Eis, Neng Huang, J. William Langston, Eli Hatchwell, Birgitt Schüle

**Affiliations:** ^1^Population Bio, Inc., New York, NY, United States; ^2^Valley Parkinson Clinic, Los Gatos, CA, United States; ^3^Department of Pathology, Stanford University School of Medicine, Stanford, CA, United States; ^4^Population Bio, UK, Begbroke, Oxfordshire, United Kingdom

**Keywords:** complex I deficiency, copy number variant, genetic risk, Ind1, mitochondrial dysfunction, NUBPL, nucleotide binding protein-like, Parkinson's disease

## Abstract

In an unbiased genome-wide screen for copy number variants (CNVs) on a cohort of Parkinson's disease (PD) patients, we identified in one patient a complex chromosomal rearrangement involving the nucleotide binding protein-like (*NUBPL*) gene on chromosome 14q12. We noted that mutations in the *NUBPL* gene had been reported as causing autosomal recessive (AR) mitochondrial Complex I (CI) deficiency in children. The precise breakpoints of the rearrangement in our PD case were found to be identical to those described in a patient with AR CI deficiency who also harbored a second pathogenic mutation in *NUBPL*. Mitochondrial dysfunction has long been considered a strong contributor to PD, and there is substantial evidence that decreased CI activity plays a central role in PD pathogenesis. We hypothesize that pathogenic *NUBPL* variants may increase the risk for PD analogous to variants in the glucosylceramidase beta (*GBA*) gene that increase the risk of developing PD in heterozygous carriers.

## Introduction

Mitochondrial dysfunction is a hallmark pathology of Parkinson's disease (PD), particularly reduced complex I (CI) activity (1, 2). Despite the biochemical evidence for impaired CI activity, and accumulation of mitochondrial DNA mutations in sporadic PD (3, 4), no pathogenic variants impacting nuclear CI genes have been reported in PD. In contrast, CI deficiency, a rare early-onset disorder, is known to occur via a recessive or X-linked mechanism for 33 nuclear-encoded CI genes. Thus, there is considerable genetic evidence that mutations impacting both alleles for CI genes result in severe phenotypes, but it is not known if heterozygous carriers of CI gene mutations may have an increased risk for late-onset neurodegenerative disorders such as PD.

We report here, for the first time, a PD patient who carries a loss-of-function variant impacting the CI gene nucleotide binding protein-like (*NUBPL*; gene aliases IND1, huInd1, C14orf127), which codes for an iron-sulfur (Fe/S) assembly protein. Since the identification of *NUBPL* as a CI deficiency gene in 2008 (5) and a clinical report in 2010 of a pediatric patient with CI deficiency due to compound heterozygous mutations in *NUBPL* (MIM 613621, 618242) (6), 17 additional patients (in 13 families) have been reported with recessive *NUBPL* disease (7–13). Our PD patient's variant, which was found in a genome-wide screen for copy number variants (CNVs), is identical to the chromosomal rearrangement found in the first reported case of *NUBPL* CI deficiency (6). We therefore propose that carriers of *NUBPL* mutations may have an increased risk for developing PD.

## Methods

The PD case described herein is from a genetic research cohort of 466 unrelated PD patients undergoing clinical care at the Parkinson's Institute and Clinical Center (Sunnyvale, CA). Written informed consent was obtained from all participants and the study was approved by El Camino Hospital's institutional review board (protocol ECH-99-22). The demographics of the cohort are: European ancestry, familial (27%) and sporadic (73%) cases, males (65%), and early onset (<50 years of age, 10%) and late onset cases (>50 years of age; 90%). A subset of the patients, including the patient reported herein, were screened for pathogenic mutations in PD genes *GBA, LRRK2, PARK7* (*DJ-1*), *PINK1, PRKN* (*PARK2*), and *SNCA* (*PARK1/4*) using the targeted 188-gene sequencing panel described by Lee et al. (14). All subjects were clinically assessed by movement disorder specialists, which included a medical history and neurological examination.

Genome-wide CNV detection on genomic DNA samples was performed using aCGH on 1 million probe microarrays (catalog design #021529) acquired from Agilent Technologies (Santa Clara, CA); detailed methods are described elsewhere (15). Briefly, raw data were generated in an ISO-certified service laboratory (Oxford Gene Technology, Oxford, UK) and analysis was performed by Population Bio. CNV data was interpreted using Population Bio's control CNV database generated on apparently healthy males and females of European ancestry. First pass assessment of potentially PD-relevant CNVs focused on large, exonic chromosomal gains or losses that were found in one or more PD cases but not in 1,000 Population Bio controls.

After filtering our patient's aCGH data to include only CNVs that were exonic and not found in our 1,000 controls, the only remaining CNV was the *NUBPL*-disrupting chromosomal rearrangement. In addition to absence in our controls, this CNV is not found in the Database of Genomic Variants. However, ClinVar reports three pediatric patients with deletion genome coordinates similar to our patient's, one is the first reported patient with recessive *NUBPL* CI deficiency (ClinVar Accession VCV000000008) (6) and the other two (ClinVar Accessions VCV000152033 and VCV000153681) were reported based on review of chromosomal microarray data for 21,698 patients that had developmental delay, intellectual disability, autism spectrum disorders, or multiple congenital anomalies (16).

**Figure 1:**
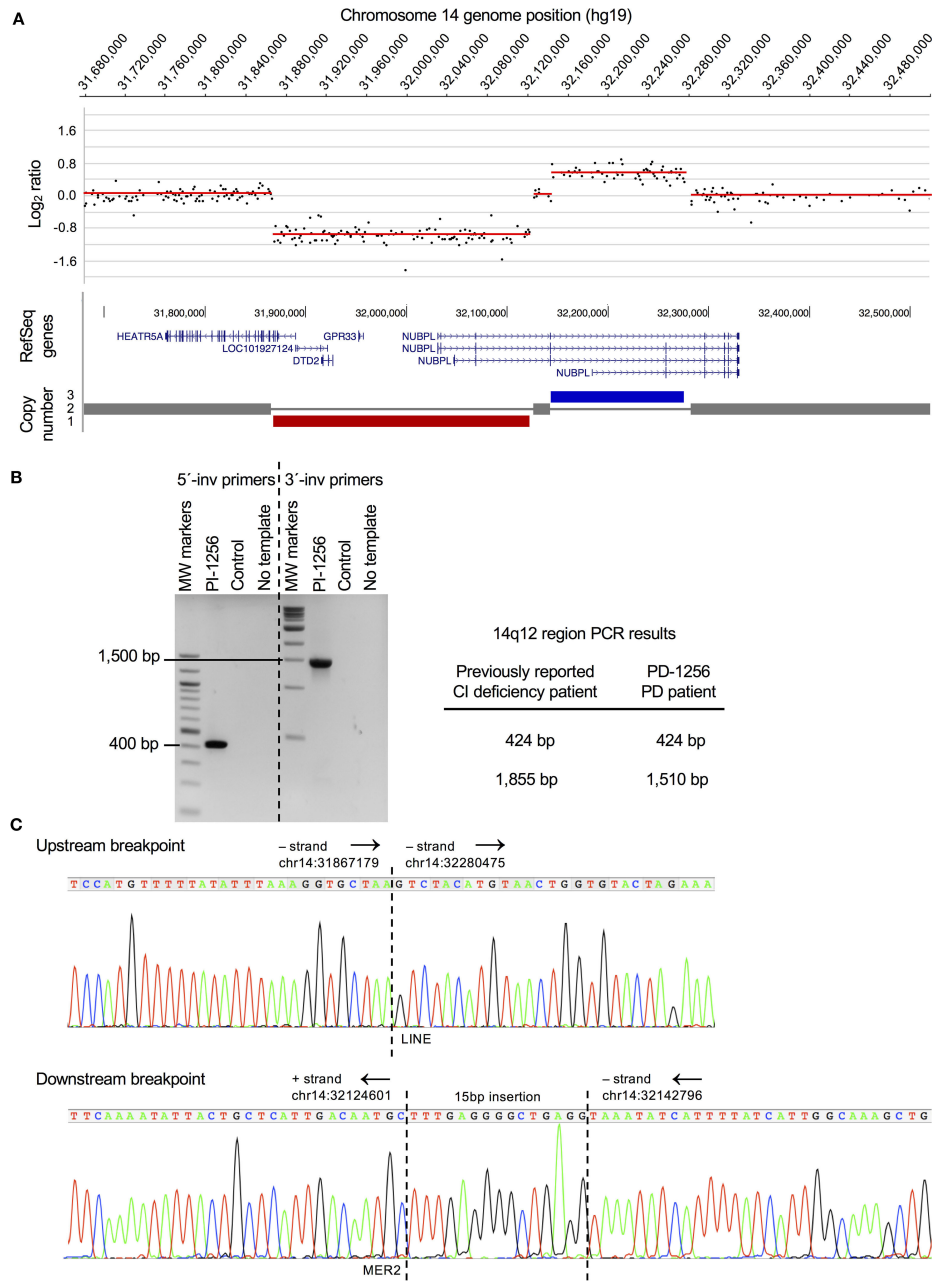
PD case with a chromosomal rearrangement disrupting CI assembly factor gene *NUBPL*. **(A)** Genome-wide CNV (aCGH) analysis revealed a complex chromosomal rearrangement in a female patient diagnosed with sporadic PD that consists of a 254Kb deletion (hg19, chr14:31867829-32121888) and 132Kb duplication (hg19, chr14:32143699-32275649). Gene annotation for the rearrangement was mapped in the UCSC genome browser (hg19) and shows *HEATR5A* and *NUBPL* are disrupted and there is a complete loss of *LOC101927124, DTD2*, and *GPR33*. Only gene dosage is shown and not the orientation of the inverted duplicated region (see **B**). Based on the microarray data, this CNV appeared to be the same as the one reported in a CI deficiency patient (6). A template switching model for the rearrangement was proposed and depicted in Tucker et al. (17) **(B)** PCR confirmation that the rearrangement detected in the sporadic PD case (PD-1256) is identical to the one previously found in a CI deficiency patient (6, 17). PCR experiments on PD-1256 were performed using previously reported PCR primers (see Methods). **(C)**. Sequence chromatograms of upstream and downstream PCR products that were generated using previously described PCR primers (17). Chromosomal positions (hg19) and orientation of the regions (black arrows) are indicated. Identical sequences were found at both breakpoints (upstream and downstream) in the PD patient as were found in the CI deficiency patient (see Methods), including the LINE and MER2 repeat elements and a 15bp insertion at the downstream breakpoint (sequence is the reverse complement of the Tucker et al. sequence).

We used the PCR primers previously described (Supplementary Data) (17) to validate the complex CNV: 5′-inversion PCR (5′-inv), forward = GTTCACCACCATACCCCAAC and reverse = GTTAACCCGCCCTTTCTCTC; 3′-inversion PCR (3′-inv), forward = AGGGGATCTGACAGTAAAAGAGG and reverse = AGAAGATACCAGGACTTTCACAG. For comparison, results for both the previously reported CI deficiency patient and our PD patient are reported (Figure 1B). The previously reported 1855bp PCR product (17) is potentially a misreported size since the breakpoint sequences and deletion and duplication sizes of this patient match those found in our PD patient. Breakpoint sequencing data (Figure 1C) in our PD patient are the same as those reported in the CI deficiency patient, based on comparison of the sequence traces [we noted an error in Tucker et al. (17) Figure 2B, which omitted a C nucleotide in the sequence listed above the upper trace data]. We verified the size of the deletion and duplication found in our PD patient by performing a BLAT analysis of each PCR product and calculating the genomic distance as follows: deletion = distance between position of breakpoint upstream of exon 1 and position of breakpoint just downstream of exon 4 (deletion removes exons 1-4); duplication = distance between position of breakpoints on either side of exon 7 (duplication/inversion of exon 7). Sanger sequencing of *NUBPL* exons and flanking sequence was performed to determine if our PD patient had any additional deleterious variants, but none were found.

## Results

We performed a genome-wide array comparative genomic hybridization (aCGH) screen of 466 PD cases and 1,000 controls to detect CNVs that could contribute to PD risk. The data were filtered for large-sized, exonic CNVs that occur in one or more PD patients but not in controls. We identified a PD patient with a complex chromosomal rearrangement, encompassing a 254 Kb deletion and a 132 Kb duplication at chromosome 14q12, that disrupts the *NUBPL* gene (Figure 1A); no other large, exonic CNVs were present in this patient. In addition to *NUBPL*, the rearrangement disrupts HEAT repeat containing 5A (*HEATR5A*) and results in complete loss of a long non-coding RNA (*LOC101927124*), D-aminoacyl-tRNA deacylase 2 (*DTD2*), and G protein-coupled receptor 33 (gene/pseudogene) (*GPR33*). We confirmed by PCR analysis (Figure 1B) and breakpoint sequencing (Figure 1C) that this complex CNV is identical to a previously described mutation in a child with CI deficiency (6, 17).

Our patient is of European ancestry with no family history of PD or parkinsonism. She resided in the United States and died at the age of 67, so we could not determine if she was related to the previously reported child with CI deficiency (6), who was reported to be from Australia (9).

Her symptoms started at age 62 with tremor of left upper and lower extremities and bradykinesia. She received clear benefit from dopamine replacement therapy; however, she quickly experienced wearing-off within a year of taking L-dopa and her symptoms progressed quickly. Cardinal symptoms of PD present upon neurological examination included asymmetric onset, bradykinesia, postural instability, rigidity, and tremor. Her speech was mildly dysarthric (dysarthria is often found in *NUBPL* CI deficiency patients) (9), she showed slight loss of facial expression, and her handwriting was severely impaired. She did not report changes in cognition or hallucinations. Autonomic symptoms included orthostatic symptoms like lightheadedness and urinary incontinence. Both her and her sister were reported to have restless legs symptoms. No dyskinesia was observed. Overall, the clinical presentation was compatible with Parkinson's disease, but differential diagnosis of multiple system atrophy was also considered because of the prominent autonomic symptoms and rather short period of symptomatic benefit from L-dopa and fast disease progression.

## Discussion

We report on the first genetic evidence of a PD case harboring a complex rearrangement in the *NUBPL* gene leading to loss of protein function. Pathogenic mutations in *NUBPL* are a cause of autosomal recessive CI deficiency in pediatric patients (MIM 613621, 618242). Our patient's mutation (Figure 1) is identical to one of the mutations reported in the first case of *NUBPL* CI deficiency (6, 17). This complex CNV (Figure 1A) results in partial loss of *HEATR5A* and *NUBPL* and full loss of *LOC101927124, DTD2*, and *GPR33*. Limited information is available for *HEATR5A* and *LOC101927124*. The *DTD2* gene is known to be involved in chiral proofreading during translation (18), and *GPR33* is linked to innate immunity and has undergone pseudogenization in humans (19). While these other genes cannot be ruled out as potential factors in PD, we consider *NUBPL* as a promising candidate PD risk gene because of strong evidence of mitochondrial CI dysfunction in PD (2).

We could not determine if our PD patient was related to the Calvo et al. patient (6), who resided in different countries (USA and Australia). While it is possible this is a recurrent rearrangement, there is no evidence of this as no differences were found (breakpoint sequences were identical, see Methods) between our PD case and the CI deficiency case (17). The ClinVar database reports two pediatric patients with similar sized deletions (see Methods), but the precise breakpoints are not known so we cannot conclude if these deletions are the same as found in our PD patient and the CI deficiency patient. If the mechanism was the same in both ClinVar cases, one would expect them to also carry a duplication but this was not reported for either patient.

Limitations of the genetic investigation methods we performed on our patient's DNA are: the aCGH microarray detects CNVs that are ~5 Kb or greater, so smaller sized CNVs (e.g., indels) or other types of structural variants (e.g., copy number neutral inversions) will not be identified; whole exome sequencing and/or whole genome sequencing was not performed on the patient's DNA. Therefore, we cannot rule out that other PD-relevant rare deleterious variants may eventually be found in our patient's genome with higher resolution methods. However, we did rule out pathogenic variants in well-established PD genes (*GBA, LRRK2, PARK7, PINK1, PRKN*, and *SNCA*) based on screening our patient's DNA with a 188-gene sequencing panel (14).

Precedents exist for genes causing early onset and severe clinical presentation when both alleles contain pathogenic mutations, and milder symptoms and later onset when only one allele is impacted by a pathogenic mutation. Notably, the association between PD and Lewy body disorders with mutations in the *GBA* gene, which causes autosomal recessive Gaucher disease (MIM 606463), has resulted in new clinical insights and drug discovery programs for PD (20, 21). Furthermore, there is growing evidence that other recessive lysosomal storage disease genes are associated with increased risk of PD in carriers of pathogenic variants (22). Analogous to the link between *GBA* heterozygous mutations and PD, we hypothesize that individuals heterozygous for *NUBPL* mutations could have an increased risk for developing PD. Given the extensive evidence for mitochondrial dysfunction — particularly reduced CI activity — in late-onset neurological disorders (2), we believe this novel finding provides a basis for the nomination of *NUBPL* as a gene that may cause or contribute to PD pathology. However, broader screening of larger cohorts of cases and controls via sequencing and copy number microarrays is warranted to support a potential association between *NUBPL* mutations and PD. Functional validation of PD-associated variants, if found, may further confirm if heterozygous carriers of *NUBPL* variants that reduce CI activity are at increased risk for PD. Finally, in order to determine if heterozygous carriers are at increased risk of PD, we suggest that it would be fruitful to examine parents and grandparents of individuals diagnosed with *NUBPL* CI deficiency.

In closing, we note that mitochondrial function is complex and involves numerous genes and environmental factors, several of which are already linked to PD (23). Large consortium driven studies have the resources to efficiently genetically validate candidate PD genes that may be causing or contributing to PD due to rare and/or common variants (24). Interestingly, a recent large study suggests that *NUBPL* and other complex I genes known to cause monogenic mitochondrial disorders are associated with PD risk and age of onset (25).

## Data Availability Statement

Publicly available data can be found here: https://www.ncbi.nlm.nih.gov/clinvar/variation/929501/ The raw data supporting the conclusions of this article will be made available by the authors, without undue reservation, to any qualified researcher. Requests to access the datasets should be directed to Eli Hatchwell (elihatchwell@populationbio.com).

## Ethics Statement

The studies involving human participants were reviewed and approved by El Camino Hospital's institutional review board (protocol ECH-99-22). The patients/participants provided their written informed consent to participate in this study. Written informed consent was obtained from the individual for the publication of any potentially identifiable images or data included in this article.

## Author Contributions

PE, EH, and BS: conception and design of the study, data acquisition, analysis, and interpretation, and wrote the manuscript. NH, JL, and BS: patient recruitment, provision of study materials, and patient clinical assessments. All authors revised/approved the manuscript.

## Conflict of Interest

PE and EH are employees of Population Bio, Inc. The remaining authors declare that the research was conducted in the absence of any commercial or financial relationships that could be construed as a potential conflict of interest.
